# CROSSTALK BETWEEN HYALURONAN AND ITS REGULATORS AFFECTS AGING

**DOI:** 10.1093/geroni/igae098.3645

**Published:** 2024-12-31

**Authors:** Kaustuv Basu, Paraskevi Heldin

**Affiliations:** Uppsala University, Uppsala, Uppsala Lan, Sweden; Uppsala University, Uppsala, Uppsala Lan, Sweden

## Abstract

The regulation of extracellular matrix (ECM) turnover is crucial in aging. Hyaluronan (HA) is a large, viscoelastic polysaccharide and a major component of ECM. It lubricates and organizes tissue structure in different body parts (e.g. skin) and orchestrates multiple cellular functions through its receptors CD44 and RHAMM and binding proteins. HA is synthesized by HA synthase (HAS). The synthesis of HA in the longest-lived rodent naked mole rat (NMR) and the extended lifespan of a transgenic mouse overexpressing the NMR-HAS2 gene illustrates the importance of HA in longevity. HA level is significantly modulated with increasing age. Thus, understanding the complex crosstalk between HA and its regulators is crucial to regulating aging. Here, we revealed a vital connection between the function of p53 and its paralogue p63 and HA biosynthesis and CD44 signaling. Previously, it was reported that p53 suppressed the expressions of CD44 and RHAMM. We identified that CD44 interacted with the apoptosis-stimulating protein of p53 (iASPP) and affected p53-mediated apoptosis. We also revealed that the mutant p53 suppressed HAS2 and -3 mRNA expression. The mutation status of endogenous p53 and the balance between p53 and p63 levels affected HAS2 and HAS3 expression differently after TGF-β1 stimulation. We noticed that the activation of p63 and Ras was essential for regulating HAS and CD44 without TGF-β1 stimulation. We observed the importance of the HA receptor in regulating aging and aging-related disease. Our study unveiled the significance of the interplay between HA and its regulators, vital in understanding the complex aging process.

